# Case Report: Multiple complications after laparoscopic cholecystectomy with perforation and spilled gallstones

**DOI:** 10.12688/f1000research.9490.2

**Published:** 2026-05-13

**Authors:** Jeanett Klubien, Dorte Winther Borgersen, Jacob Rosenberg, Hans-Christian Pommergaard

**Affiliations:** 1 Center for Perioperative Optimization, Department of Surgery, Herlev Hospital, Herlev, Denmark; 2 Faculty of Health and Medical Sciences, University of Copenhagen, Copenhagen, Denmark; 3 Department of Surgery, Herlev Hospital, Herlev, Denmark; 4 Department of Surgery, Hvidovre Hospital, Hvidore, Denmark

**Keywords:** Laparoscopic cholecystectomy, spilled gallstones, lost gallstones, abscess, fistula, empyema, case report

## Abstract

**Abstract:**

**Introduction:**

Perforation of the gallbladder is a benign and common complication during laparoscopic cholecystectomy. However, it may result in stone spilling, which potentially can lead to serious postoperative complications.

**Case report:**

A 70-year-old male underwent laparoscopic cholecystectomy for acute cholecystitis. The procedure was complicated by perforation of the gallbladder and spilling of gallstones. More than a year after the procedure, the patient developed subcutaneous abscesses containing some of the spilled stones, a computed tomography revealed a complex intraabdominal and intrathoracic fistula with communication from the abdominal cavity to pleura and ultrasonic imaging found a lost gallstone in the thorax. After two years, the patient developed pleural empyema and sepsis secondary to the condition. Presently, the patient awaits surgery for the fistula and empyema.

**Conclusion:**

Proper care should be taken to avoid stone spilling during laparoscopic cholecystectomy. However, if perforation and stone spilling occur, all visible stones should be removed during the procedure and the complication should be noted in the medical records. Furthermore, the patient should be thoroughly informed. This may help accelerate diagnosis if the patient later suffers from a complication related to lost stones.

## Introduction

Perforation of the gallbladder during laparoscopic cholecystectomy (LC) is a well-known and common complication (8–40%) ^1^ that may lead to intraabdominal spilling of gallstones and some of the spilled stones may not be retrieved despite all efforts. The incidence of lost stones during LC is less frequent and varies in the literature from 0.1 to 20%. ^1^^–^^3^ Although considered a benign complication, it is reported that 0.03–8.5% of the lost stones will lead to a postoperative complication. ^2^^,^^3^

We present a case of multiple complications after perforation of the gallbladder and subsequent stone spilling during LC. This case report is reported according to the CARE statement. ^4^

## Case report

A 70-year-old Caucasian male, with a medical history of hypertension, was admitted in March 2014 after four days of diffuse abdominal pain and fever up to 39°C. A computed tomography (CT) scan identified multiple gallstones in an inflamed gallbladder. To verify the diagnosis, abdominal ultrasonic imaging confirmed multiple gallstones and thickening of the gallbladder wall as signs of acute cholecystitis. The patient underwent acute LC with the intraoperative finding of a severely inflamed gallbladder. In addition, the procedure was complicated by perforation of the gallbladder and gallstones were spilled. The gallbladder was removed using an endoscopic bag after complete dissection to prevent further stone spilling and all visible stones were removed. Lastly, the peritoneal cavity was irrigated with saline to retrieve any additional gallstones. The complication was noted in the medical records.

**Figure 1.**
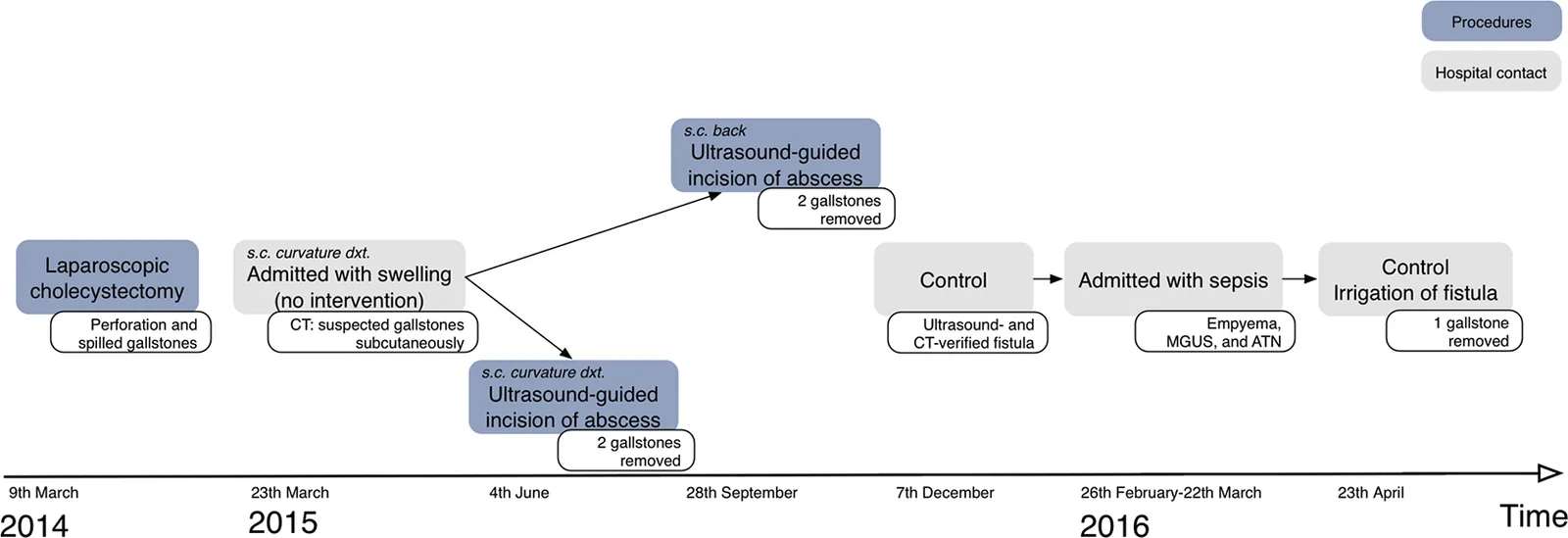
Timeline. An overview of the patient's hospital contacts and procedures after the laparoscopic cholecystectomy. *s.c. subcutaneous, dxt. dexter, CT computed tomography, MGUS monoclonal gammopathy of undetermined significance, ATN acute tubular necrosis.*

One year after the procedure, the patient was admitted with tenderness in the right upper quadrant. A CT was performed and showed a swelling in the upper right part of the abdominal wall and between the liver and the lower lobe of the right lung with calcifications at both sites assumed to be lost gallstones ( Figure 1). The patient did not receive any treatment for the swellings.

**Figure 2.**
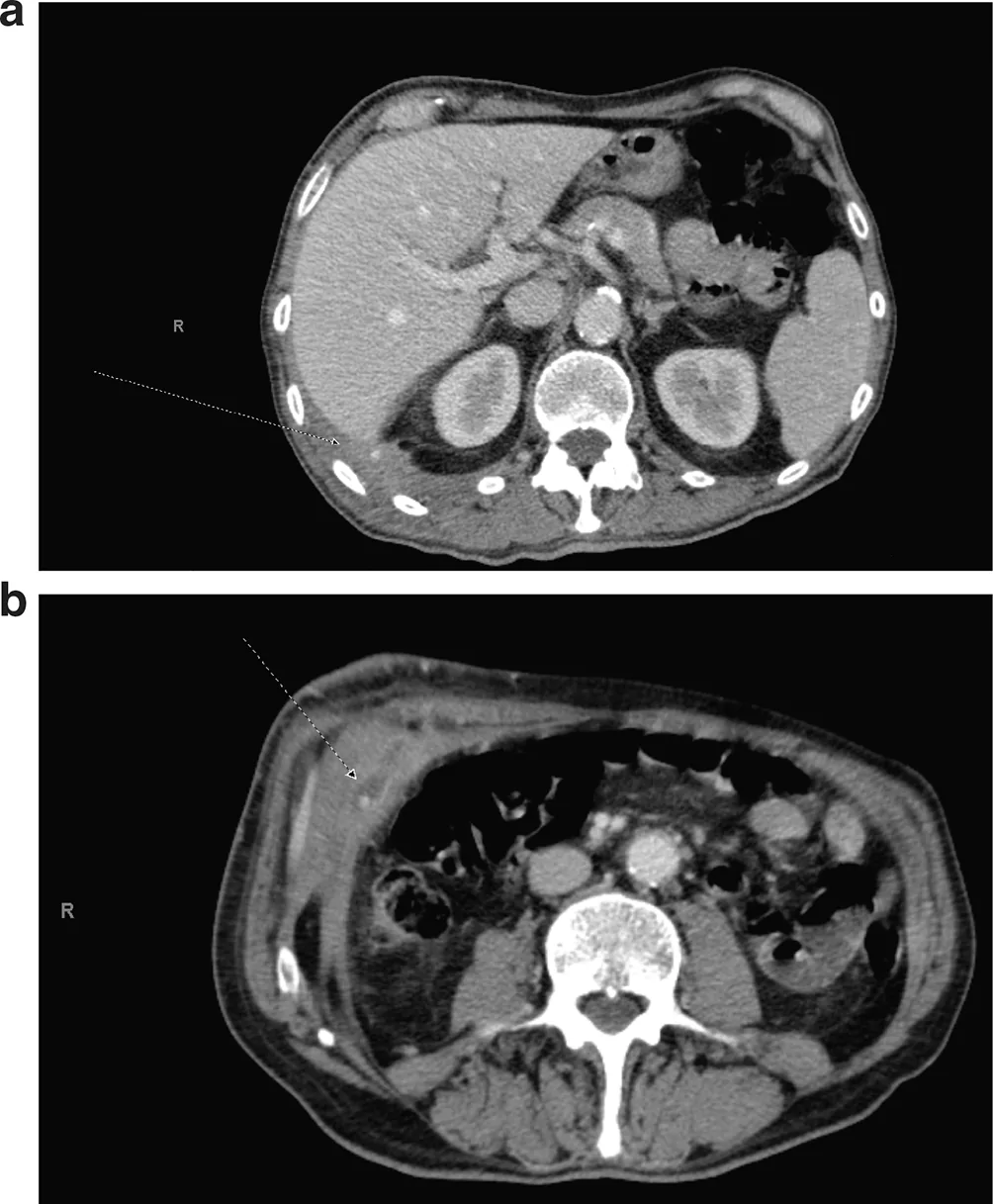
Abdominal computed tomography. An abdominal computed tomography showing spilled gallstones at different levels 15 months after the laparoscopic cholecystectomy (dotted arrows). (a) Shows a gallstone behind the liver and (b) shows a gallstone in the abdominal wall.

During the period between 15 and 18 months following the LC, the patient returned to the hospital two times due to subcutaneous abscesses below the right rib curvature and the right side of the lower back. The suspected lost gallstones were assumed to have migrated to the subcutaneous tissue causing abscess formation. The diagnosis was confirmed by CT and compared with the previous CT ( Figure 2). Both abscesses were located deep in the subcutaneous tissue and due to location and size, these were treated with ultrasound-guided incision and drainage. Additional information regarding bacterial culture or antibiotic treatment were, unfortunately, not retrieved from the patient’s medical record. During these procedures, four gallstones were located and removed from the abscess cavities. Afterwards the patient was followed as an outpatient because of daily secretion from the abscess cavity on the patient’s back. Because of the unhealed abscess cavity, CT and ultrasound scans were performed 18 months after the LC. The CT revealed a complex intraabdominal and intrathoracic fistula with external opening in the lower right side of the back with communication to pleura. The ultrasonic imaging revealed a lost gallstone in the lower right side of thorax. The fistula was treated conservatively with drainage.

In February 2016, the patient was admitted to the hospital because he had developed sepsis and pleural empyema secondary to the condition. The patient had a short stay at the intensive care unit and was discharged from the hospital after one month. During this month, the patient developed monoclonal gammopathy and acute tubular necrosis due to the infection in the fistula. After hospitalization, the fistula was rinsed daily with saline solution and during one of these procedures another gallstone was excavated. Presently, the patient awaits surgery for the fistula and empyema.

## Discussion

This case is an example of serious complications caused by spilled gallstones. Migration of lost stones, as in this case, can cause both local and systemic complications. However, stone spillage is unavoidable in some patients despite precautionary measures.

The spilled stones may be harmless, but efforts should be made during the procedure to locate and remove all stones to prevent future local and systemic complications. The postoperative complications due to lost gallstones may develop weeks to several years after the primary procedure and are not necessarily located in the right upper quadrant. ^2^^,^^5^^,^^6^ Together with a lack of awareness or documentation in the medical records, this may contribute to a delayed diagnosis of a stone complication. However, delayed diagnosis may also be due to the fact that some gallstones are not visible on CT. Predisposing factors for complications of the spilled gallstones include older age, male sex, perihepatic localization of lost stones, acute cholecystitis, spilling of pigment stones compared with cholesterol stones, multiple stones (>15 stones), and large stone size (> 1.5 cm). ^1^

It is not mandatory to convert to open surgery for retrieving stones after perforation has occurred during LC, ^3^^,^^6^ due to a subsequent low incidence of severe postoperative complications ^2^^,^^3^ and since conversion to open surgery is associated with a higher rate of systemic complications compared with laparoscopic surgery. ^3^ In this case report, the surgeon chose not to convert to open surgery to look for more lost gallstones, which goes well in hand with the recommendations found in the literature. ^3^^,^^6^ However, proper care should be taken to avoid stone spilling and thereby possible postoperative complications. All visible stones should be removed during the laparoscopic procedure and the gallbladder should be retrieved in an endoscopic bag upon dissection to prevent further stone spilling when a perforation has occurred. ^7^ In this case, the gallstones were found on CT before complications developed. Perhaps, the abscesses and fistula could have been avoided if the stones had been removed when they were discovered.

In conclusion, stone spillage is an unavoidable and well-known problem to LC. If perforation and stone spillage occur, it should be noted in the medical records and the patient should be thoroughly informed about the lost stones and their possible postoperative complications. This may help the clinicians and accelerate the diagnosis if the patient later on suffers from a complication due to lost stones.

## Consent

Written informed consent was obtained from the patient for publication of this case report and any accompanying images and/or other details that could potentially reveal the patient’s identity.

## Data Availability

No data availability associated with the manuscript. JK, DW, JR, and HCP conceived the study. JK and HCP prepared the first draft of the manuscript. DWB, JR and HCP did the revision and all authors have read and approved the final version of the manuscript.
