# Supplementary material for: Case Report: Multiple complications after laparoscopic cholecystectomy with perforation and spilled gallstones
Source: F1000Res. 2026 Jul 9;5:2322. Originally published 2016 Sep 14. [Version 3] doi: 10.12688/f1000research.9490.3 (PMC13550883; doi:10.12688/f1000research.9490.3)
Supplement: Peer review history

# Reviewer report: Reviewer response for version 3

**DOI:** 10.5256/f1000research.205380.r499873

**Published:** 2026-07-10

**Authors:** Emre Gonullu

**Affiliations:** 1 Sakarya University Training and Research Hospital, Sakarya, Turkey

Thank you for your careful and comprehensive responses to the reviewer comments. The revised manuscript has been substantially improved. The discussion now provides a clearer rationale for the conservative management approach, the proposed mechanism of fistula formation has been expanded appropriately, and the lack of microbiological data has been clearly acknowledged as a limitation. Although additional imaging was unavailable, the explanation provided is satisfactory. I have no further comments and believe the manuscript is suitable for indexing .

Are enough details provided of any physical examination and diagnostic tests, treatment given and outcomes?

Partly

Is the case presented with sufficient detail to be useful for other practitioners?

Yes

Is sufficient discussion included of the importance of the findings and their relevance to future understanding of disease processes, diagnosis or treatment?

Yes

Is the background of the case’s history and progression described in sufficient detail?

Yes

Reviewer Expertise:

Gastrointestinal Surgery

I confirm that I have read this submission and believe that I have an appropriate level of expertise to confirm that it is of an acceptable scientific standard.

## References

[1] : Retained Abdominal Gallstones After Laparoscopic Cholecystectomy. *Surgical Laparoscopy, Endoscopy & Percutaneous Techniques* .2015;25(2) : 10.1097/SLE.0000000000000105 97-99 10.1097/SLE.0000000000000105 25304733

# Reviewer report: Reviewer response for version 2

**DOI:** 10.5256/f1000research.198042.r493690

**Published:** 2026-06-23

**Authors:** Emre Gonullu

**Affiliations:** 1 Sakarya University Training and Research Hospital, Sakarya, Turkey

A rare and educational case is presented describing multiple delayed complications caused by spilled gallstones after laparoscopic cholecystectomy, including recurrent subcutaneous abscesses, a thoracoabdominal fistula, pleural empyema, and sepsis. The manuscript highlights an important yet often underestimated complication of laparoscopic cholecystectomy and provides a valuable reminder to prevent stone spillage, document intraoperative events, and inform patients about potential long-term consequences.

 The clinical history and chronological progression of events are generally well described, and the included imaging findings and timeline help readers follow the patient’s course. The manuscript also provides a useful review of the literature regarding the incidence and potential consequences of retained gallstones. The case is clinically relevant because it demonstrates that complications may occur long after the index procedure and may present in unusual anatomical locations.

 However, several aspects of the report could be strengthened. Details regarding microbiological evaluation are lacking. Information about bacterial cultures obtained from the abscesses, isolated organisms, and antibiotic treatment would significantly improve the scientific value of the report. If these data are unavailable, this limitation should be emphasized more clearly. In addition, the rationale for management decisions deserves further discussion. Retained gallstones were identified on imaging before the development of the most severe complications, yet no intervention was undertaken at that stage. A more detailed explanation of the decision-making process and an examination of alternative management strategies would be valuable to readers.

 The discussion would also benefit from a more comprehensive exploration of the mechanism of fistula formation and pleural extension. Although chronic inflammation related to retained gallstones is a plausible explanation, the potential anatomical pathways and contributing factors could be discussed in greater detail. Furthermore, additional imaging demonstrating the fistulous tract, if available, would strengthen the report and increase its educational impact.

 Overall, this is a noteworthy and publishable case report with clear clinical relevance. The manuscript would benefit from minor revisions addressing the issues above, particularly clarification of microbiological data, treatment details, and the discussion of management decisions and fistula development. Subject to these revisions, the article is suitable for indexing.

Are enough details provided of any physical examination and diagnostic tests, treatment given and outcomes?

Partly

Is the case presented with sufficient detail to be useful for other practitioners?

Yes

Is sufficient discussion included of the importance of the findings and their relevance to future understanding of disease processes, diagnosis or treatment?

Yes

Is the background of the case’s history and progression described in sufficient detail?

Yes

Reviewer Expertise:

Gastrointestinal Surgery

I confirm that I have read this submission and believe that I have an appropriate level of expertise to confirm that it is of an acceptable scientific standard, however I have significant reservations, as outlined above.

# Response

**Published:** 2026-07-01

**Authors:** Jeanett Klubien

**Affiliations:** Herlev Hospital, Denmark

**Concerning the revised version of the manuscript: Case Report: Multiple complications after laparoscopic cholecystectomy with perforation and spilled gallstones (article number: 9490)**

*We sincerely thank the reviewer for a thorough assessment of our manuscript and appreciate the valuable suggestions, which have improved its quality and clarity.*

 1. Details regarding microbiological evaluation are lacking. Information about bacterial cultures obtained from the abscesses, isolated organisms, and antibiotic treatment would significantly improve the scientific value of the report.

***We fully agree with the reviewer. Unfortunately, detailed microbiological results and information regarding antibiotic treatment cannot be retrieved from the patient's medical records. We have added the following in the Discussion section to acknowledge this as a limitation:***

***“A limitation of this case report is that microbiological findings, including bacterial cultures, isolated organisms, and details of antibiotic treatment, could not be retrieved from the patient's medical records. These data would have provided additional and relevant insight into the infectious course and management of this patient.”***

 2. The rationale for management decisions deserves further discussion. Retained gallstones were identified before the development of the most severe complications, yet no intervention was undertaken at that stage.

***We thank the reviewer for this observation, and we have added the text below to the Discussion section:***

***“In most cases, retained gallstones remain clinically silent. In this case, the retained gallstones were identified on CT one year after laparoscopic cholecystectomy; however, as the patient was asymptomatic, conservative management was chosen in accordance with current practice, given the low incidence of clinically significant complications and the morbidity associated with reoperation. With hindsight, earlier intervention may have altered the subsequent clinical course.”***

 3. The discussion would benefit from a more comprehensive exploration of the mechanism of fistula formation and pleural extension.

***We have revised the Discussion section to further elaborate on the proposed mechanism. The following has been added to the text:***

***“Retained gallstones in the peritoneal cavity may over time cause a local inflammatory reaction, leading to abscess formation. Persistent inflammation may result in erosion into adjacent tissues and subsequent fistula formation. This is a rare complication after LC, but has been reported previously, including bronchobiliary and colonic fistula formation.** ^5,6^”*

 4. Additional imaging demonstrating the fistulous tract, if available, would strengthen the report.

***We agree that additional imaging would enhance the value of the report. Unfortunately, no further imaging is available**.*

# Reviewer report: Reviewer response for version 1

**DOI:** 10.5256/f1000research.10221.r16707

**Published:** 2016-10-10

**Authors:** Tatsuhiro Masaoka

**Affiliations:** 1 Department of Internal Medicine, Keio University School of Medicine, Tokyo, 160-8582, Japan

Authors reported a case complicated with stone spillage after laparoscopic cholecystectomy (LC). Perforation of the gallbladder during LC is a common complication and the incidence of lost stones during LC is not so rare. However it is rare that lost stones lead such a severe postoperative complication. So I think this paper is very interesting. However the authors had better concern the following points.

1. Case report session is well written. Authors summarize the long progression in compact. I would like to know at one year after procedure how do authors indicate treatment options to patients and his family. Authors described a CT just showed swelling around calcification. However the patient was conscious of abdominal tenderness, and the cause of tenderness is assumed to be lost gallstone. I think operation must be considered as the best treatment at this point. Authors should discuss more about this point. During the period between 15 and 18 months following the LC, subcutaneous abscesses were formed. The patient was treated with ultrasound-guided incision and drainage. Did authors take bacterial culture? How about antibacterial treatment? Proper antibacterial treatment is important for preventing for progression of diseases. If done, authors should mention detail antibacterial treatment such as choices, doses, duration etc.
2. The discussion session is also well written. However authors did not mentioned fistula with communication to pleura. I would like to know why the patient developed fistula. What is the main factor of fistulization? The position of spilled gallstone? Inadequate treatment for abscess? Just after LC, did authors take CT? Of course operation for removing spilled gallstone might be best choice for preventing fistulization when the gallstone was found. Is there any other choice avoiding fistula with communication to pleura? Authors should discuss about this point.

Reviewer Expertise:

NA

I confirm that I have read this submission and believe that I have an appropriate level of expertise to confirm that it is of an acceptable scientific standard, however I have significant reservations, as outlined above.

# Response

**Published:** 2026-01-20

**Authors:** Jeanett Klubien

**Affiliations:** Herlev Hospital, Denmark

1. Case report session is well written. Authors summarize the long progression in compact. I would like to know at one year after procedure how do authors indicate treatment options to patients and his family. Authors described a CT just showed swelling around calcification. However the patient was conscious of abdominal tenderness, and the cause of tenderness is assumed to be lost gallstone. I think operation must be considered as the best treatment at this point. Authors should discuss more about this point. During the period between 15 and 18 months following the LC, subcutaneous abscesses were formed. The patient was treated with ultrasound-guided incision and drainage. Did authors take bacterial culture? How about antibacterial treatment? Proper antibacterial treatment is important for preventing for progression of diseases. If done, authors should mention detail antibacterial treatment such as choices, doses, duration etc.

*Response: Thank you for this thoughtful comment. Unfortunately, detailed information regarding discussions of treatment options with the patient and family is not available. Likewise, no additional data regarding bacterial cultures or antibiotic therapy were available. We agree that microbiological evaluation and appropriate antibiotic treatment are important considerations in such cases, and we acknowledge this as a limitation of the present report.*

  

1. The discussion session is also well written. However authors did not mentioned fistula with communication to pleura. I would like to know why the patient developed fistula. What is the main factor of fistulization? The position of spilled gallstone? Inadequate treatment for abscess? Just after LC, did authors take CT? Of course operation for removing spilled gallstone might be best choice for preventing fistulization when the gallstone was found. Is there any other choice avoiding fistula with communication to pleura? Authors should discuss about this point

*Response: Thank you for this comment. The exact mechanism of the fistulation remains unclear, but it might have been driven by chronic inflammation originating from the retained gallstones. The anatomical location and repeated abscess formation likely contributed. While surgical removal of retained gallstones could have reduced the risk of fistula formation once* *identified, the decision must be balanced against operative risks, particularly in patients with mild or nonspecific symptoms.*

# Reviewer report: Reviewer response for version 1

**DOI:** 10.5256/f1000research.10221.r16285

**Published:** 2016-09-26

**Authors:** Gabriel Sandblom

**Affiliations:** 1 Department of Clinical Sciences, Intervention and Technology, Karolinska Institute, Stockholm, Sweden

The report describes a rare case where spilled gallstones following laparoscopic cholecystectomy resulted in fistulas to the subcutaneous tissue as well as to the pleura.  Albeit an uncommon late complication from spilled gallstones, the report is an important reminder of the hazards of gallstone surgery and an argument for careful surgical technique in laparoscopic cholecystectomy for acute cholecystitis. Even if there is no high risk of developing severe complications such as those described in the report, it should be kept in mind when performing laparoscopic cholecystectomy. Granulomas due to gallstones left in the abdominal cavity is probably an overseen complication following gallstone surgery that may cause persisting pain.

 Comments:

1. Did the report from the cholecystectomy include more detailed description of the spilled gallstones? Where the gallstones left in the trochar site (which could explain the subcutaneous fistlulas)? Where any attempts taken to extract the stones?
2. Were any bacterial cultures taken? The aggressive local growth, with formation of abscesses as well as fistulas raised the suspicion of uncommon pathogens (e.g. Salmonella Virchow or Clostridium perfringens)
3. In the discussion “In this case, the gallstones were found on CT before complications developed.  Perhaps, the abscesses and fistula could have been avoided if the stones had been removed when they were discovered.”. Do the authors suggest a repeated laparoscopic procedure, even in asymptomatic patients, in order to remove spilled gallstones? This seems like a slightly hyperbolic recommendation from this single experience. A laparoscopic exploration aimed at discovering gallstones overseen at the first procedure may hardly be expected to reduce the risk of late complications.

Reviewer Expertise:

NA

I confirm that I have read this submission and believe that I have an appropriate level of expertise to confirm that it is of an acceptable scientific standard, however I have significant reservations, as outlined above.

# Response

**Published:** 2026-01-20

**Authors:** Jeanett Klubien

**Affiliations:** Herlev Hospital, Denmark

1. Did the report from the cholecystectomy include more detailed description of the spilled gallstones? Where the gallstones left in the trochar site (which could explain the subcutaneous fistlulas)? Where any attempts taken to extract the stones?

*Response: Thank you for this comment. The operative report did not have a more detailed description than already presented in the manuscript. All visible gallstones were removed intraoperatively, and the peritoneal cavity was irrigated with saline to retrieve any additional gallstones; no stones were left intentionally.*

  

1. Were any bacterial cultures taken? The aggressive local growth, with formation of abscesses as well as fistulas raised the suspicion of uncommon pathogens (e.g. Salmonella Virchow or Clostridium perfringens)

*Response: Thank you for this insightful comment. Unfortunately, information regarding bacterial cultures, including identification of specific pathogens, was not available.*


*  *

1. In the discussion “In this case, the gallstones were found on CT before complications developed.  Perhaps, the abscesses and fistula could have been avoided if the stones had been removed when they were discovered.”. Do the authors suggest a repeated laparoscopic procedure, even in asymptomatic patients, in order to remove spilled gallstones? This seems like a slightly hyperbolic recommendation from this single experience. A laparoscopic exploration aimed at discovering gallstones overseen at the first procedure may hardly be expected to reduce the risk of late complications.

*Response: We appreciate the comment and fully agree that a recommendation should not be based on a single case. We do not advocate for repeated laparoscopic exploration in asymptomatic patients to retrieve lost gallstones. We intended to reflect on this specific case, where calcifications presumed to be gallstones were identified on CT imaging. At that time, the patient’s symptoms were mild, and the potential risks of surgical intervention were considered to outweigh the possible benefits.*
